# Associations between the consumption of carbonated beverages and periodontal disease: The 2008–2010 Korea national health and nutrition examination survey

**DOI:** 10.1097/MD.0000000000004253

**Published:** 2016-07-18

**Authors:** In-Seok Song, Kyungdo Han, Youngkyung Ko, Yong-Gyu Park, Jae-Jun Ryu, Jun-Beom Park

**Affiliations:** a Department of Oral and Maxillofacial Surgery, Korea University Anam Hospital; b Department of Biostatistics; c Department of Periodontics, College of Medicine, The Catholic University of Korea; d Department of Prosthodontics, Korea University Anam Hospital, Seoul, Republic of Korea.

**Keywords:** carbonated beverages, dentition, epidemiology, nutrition surveys, oral health

## Abstract

Consumption of carbonated beverages was reported to be associated with obesity and other adverse health consequences. This study was performed to assess the relationship between the consumption of carbonated beverages and periodontal disease using nationally representative data.

The data from the Korea National Health and Nutrition Examination Survey conducted between 2008 and 2010 were used; the analysis in this study was confined to a total of 5517 respondents >19 years old who had no missing values for the consumption of carbonated beverages or outcome variables. The community periodontal index greater than or equal to code 3 was defined as periodontal disease.

The odds ratios of the percentage of individuals with periodontal treatment needs tended to increase with the consumption of carbonated beverages. Adjusted odds ratios and their 95% confidence intervals adjusted for various factors including age, sex, body mass index, smoking, drinking, exercise, metabolic syndrome, frequency of tooth brushing per day, use of secondary oral products, dental checkup within a year, consumption of coffee of the individuals with the consumption of carbonated beverages once or less per month, once or less per week and twice or more per week were 1.109(0.804,1.528), 1.404(1.035,1.906), and 1.466(1.059,2.029), respectively. A subgroup analysis revealed that in individuals with body mass index < 25 or waist circumference < 90 cm for males or < 80 cm for females, the prevalence of periodontal disease increased with higher consumption of carbonated beverages (*P* for trend < 0.05).

Consumption of carbonated beverages was positively associated with the risk of periodontal disease in Korean adults. In a subgroup analysis, the individuals consuming carbonated beverages with body mass index < 25 or waist circumference < 90 cm for males or < 80 cm for females were more likely to have periodontal disease. Consumption of carbonated beverages may be considered to be an independent risk indicator for periodontal disease and periodontal health of nonobese individuals may benefit from reduction of carbonated beverage consumption.

## 1 Introduction

The frequency of consumption of carbonated beverages remains high in the United States,^[1]^ and South Korea is following the global nutrition transition toward greater consumption of sugar-sweetened beverages.^[2]^ High consumption of carbonated beverages may lead to health concerns, as carbonated beverages usually contain significant amounts of sugar.^[3]^ Sugar-sweetened carbonated beverage consumption was reported to be correlated with body mass index and waist circumference in school children.^[4]^ Consequently, restricting soft drink consumption was suggested as part of an initiative to prevent childhood obesity.^[5]^ Carbonated soft drink consumption was inversely related to bone mineral density in adolescent girls.^[6]^ Similarly, carbonated beverage consumption and bone fractures were associated in teenage girls, and this association between consumption of carbonated beverages and bone fractures was more evident in physically active girls.^[7]^ Increased consumption of sugars has been identified as a potential risk factor in an increase in caries activity,^[3]^ and especially high consumption of carbonated soft drinks by young children is a risk indicator for dental caries in the primary dentition.^[8]^

Periodontitis is a chronic inflammatory disease of periodontal tissue affecting nearly 50% of the general population,^[9,10]^ and it is well known to be not only a local phenomenon but also to be connected with systemic diseases.^[11]^ It was hypothesized that there was no significant association between the amount consumption of carbonated beverages and periodontal disease. Thus, this study was performed to assess the relationship between the consumption of carbonated beverages and periodontal treatment needs using nationally representative data.

## 2 Methods

### 2.1 Overview of the survey and participants

This study was based on data derived from the Korea National Health and Nutrition Examination Survey (KNANES), which was conducted between 2008 and 2010 by the Division of Chronic Disease Surveillance under the Korea Centers for Disease Control and Prevention and the Korean Ministry of Health and Welfare.^[12]^ The KNHANES is a nationwide survey of noninstitutionalized civilians and it is conducted annually by using a complex, stratified, multistage probability-cluster design. The sample weights were used to calculate all statistics of this survey. To represent the Korean population with sample participants, sample weights were created, considering survey nonresponse, complex survey design, and poststratification.

Initially, a total of 29,235 individuals participated in the KNHANES survey. The analysis in this study was confined to a total of 5517 respondents (aged 19–39 years) who had no missing values for the consumption of carbonated beverages or outcome variables. Consumption of carbonated beverages was calculated based on the survey. All participants in the survey signed an informed consent form prior to participation. This survey was reviewed and approved by the Institutional Review Board of the Korea Centers for Disease Control and Prevention. The Institutional Review Board at the Catholic University of Korea approved of this study (KC14EISI0636).

### 2.2 Sociodemographic and lifestyle variables

All individuals were asked about sociodemographic variables by trained interviewers. The education level was categorized based on the status whether or not the participant had graduated from high school. When monthly income was lower than $1092.40 USD, the monthly household income was designated as the lowest quartile after adjusting for the number of family members. The consumption of alcohol was calculated using the results of the survey considering the amount of alcoholic beverages and frequency of consumption, as reported previously: nondrinker, light-to-moderate drinker (1–30 g/day), and heavy drinker (> 30 g/day).^[13]^ Smoking status was defined according to self-reported cigarette use and based on current smoking habits: non-smoker, ex-smokers, and current smokers.^[14]^ The individuals were regarded as regular physical exercisers if the participant were engaged in a moderate fitness activity on a regular basis for 30 minutes or longer at a time at least 5 times per week, or for 20 minutes or longer at a time at least 3 times per week in a vigorous fitness activity.^[15]^

### 2.3 Measurements and classification of variables

Anthropometric measurements were performed by trained staff members. Body weight and height were measured with the subject wearing light clothing. The body mass index (BMI) was calculated using the formula: BMI = weight (kg)/height (m^2^). Waist circumference was measured at the level midway between the costal margin and the iliac crest at the end of a normal expiration.

Concentrations of serum fasting plasma glucose, total cholesterol, triglycerides, high-density lipoprotein-cholesterol, and white blood cell count were measured from the blood sample collected from the antecubital vein after fasting for >8 hours. Metabolic syndrome was defined based on the previous report.^[16]^ Three or more of the following criteria must be fulfilled in order to be diagnosed with metabolic syndrome: waist circumference ≥ 90 cm for men and ≥ 80 cm for women; fasting triglycerides ≥ 150 mg/dL or the use of lipid-lowering medication; high-density lipoprotein cholesterol < 40 mg/dL in men and < 50 mg/dL in women or use of medication; blood pressure ≥ 130/85 mm Hg or the use of hypertension medication; and fasting blood glucose ≥ 100 mg/dL or the current use of diabetes medication.

### 2.4 Periodontal treatment needs and oral health behaviors

The frequency of daily tooth brushing was measured by the total number of times the teeth were brushed per day. Use of secondary oral products, dental checkup within a year, self-reported oral status, chewing ability, and speech were evaluated.

The World Health Organization community periodontal index (CPI) was used to assess periodontal treatment needs and defined periodontal disease as CPI greater than or equal to code 3. A CPI score of code 3 indicates that at least 1 site had a > 3.5 mm pocket in the 10 specific index teeth (17, 16, 11, 26, 27, 36, 37, 31, 46, and 47). The mouth was divided into sextants. A CPI probe (PWHO, Osung MND, Seoul, South Korea) with a 0.5 mm ball tip was used. A sextant was examined only if there were 2 or more teeth present that were not scheduled for extraction. If no index teeth were present in a sextant qualifying for examination, all remaining teeth were examined and the highest score was recorded as the score for that sextant.

### 2.5 Statistical analyses

The data are presented as means ± standard errors for continuous variables and as proportions (standard errors) for categorical variables. If necessary, logarithmic transformations were performed for variables with skewed distributions. A chi-square test for categorical variables or an independent *t* test for continuous variables was performed to assess the differences in characteristics. Multiple logistic regression analyses were used to assess the associations of periodontal treatment needs and consumption of carbonated beverages. The model was adjusted for age, sex, body mass index, smoking, drinking, exercise, metabolic syndrome, frequency of tooth brushing per day, use of secondary oral products, dental examination within a year, and consumption of beer and coffee. All statistical analyses were performed with the SAS software (ver. 9.2 for Windows; SAS Institute; Cary, NC). A *P* value < 0.05 was considered to indicate statistical significance.

## 3 Results

Table 1 describes baseline characteristics of the study individuals according to the presence of periodontal treatment needs. The mean age, body mass index, and waist circumference were significantly higher in participants with periodontal disease. Current smokers, heavy drinkers, and individuals from the lowest income quartile were more likely to have periodontal disease. Those with a high school degree or greater and those doing regular exercise were less likely to have periodontal disease. Use of secondary oral products and the number of dental visits were significantly lower in participants with periodontal disease.

**Table 1:**
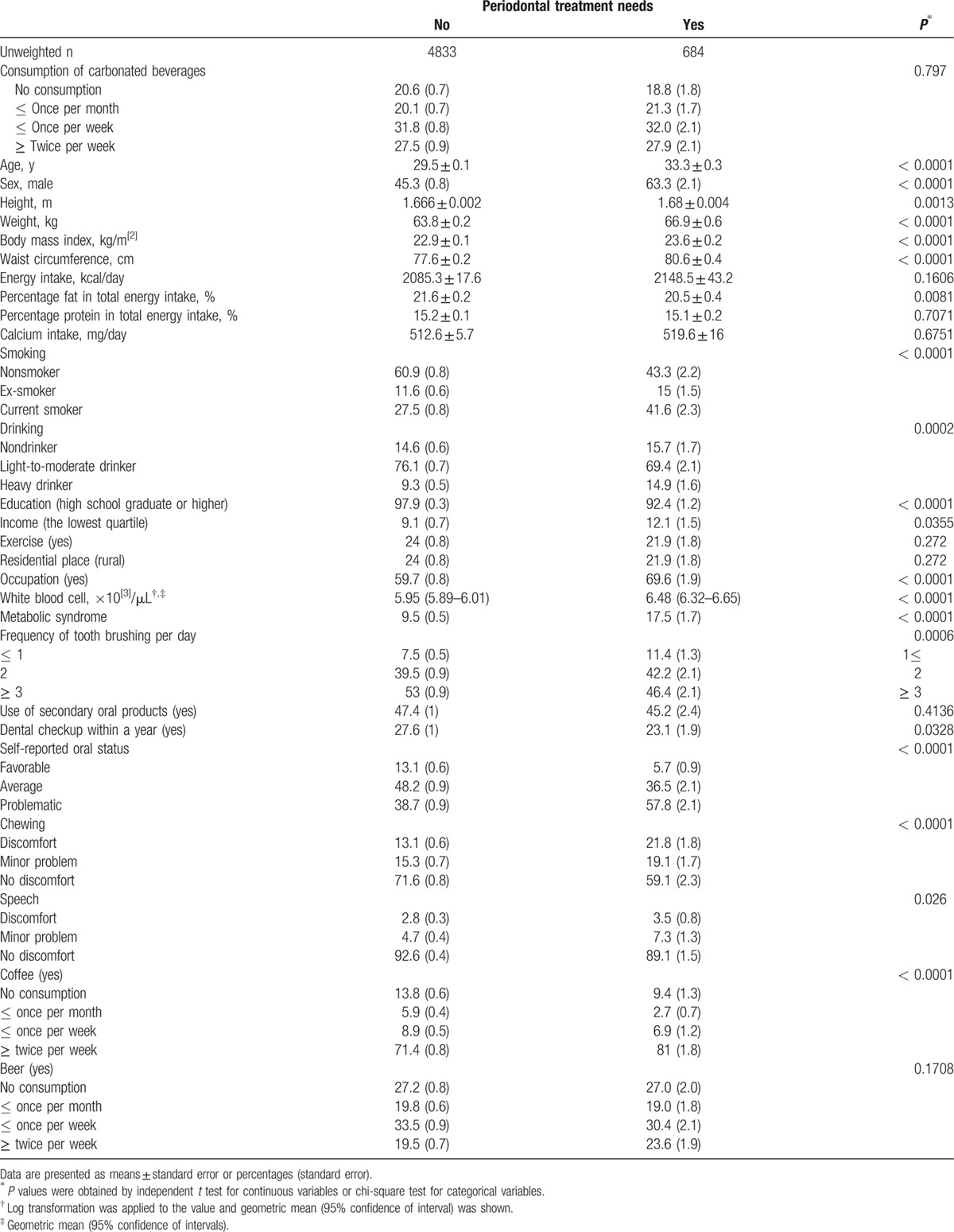
Baseline characteristics of study individuals according to periodontal disease and the number of natural teeth.

**Figure 1:**
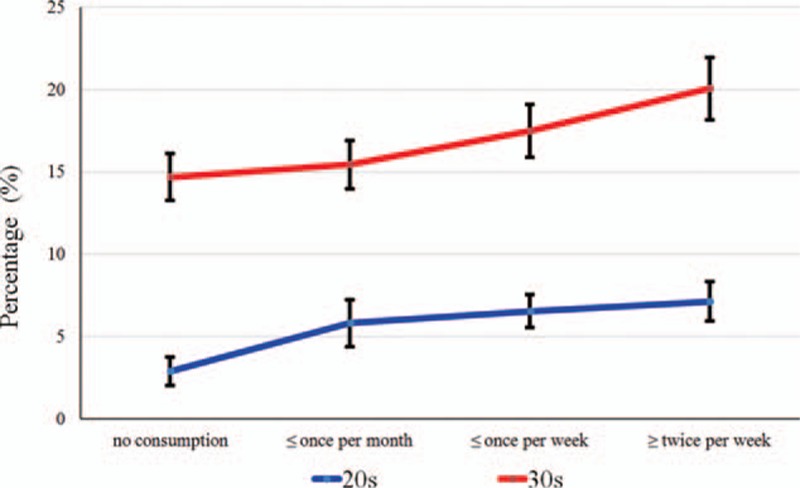
The percentage and standard error of the individuals categorized by the presence of periodontal disease.

Figure 1 shows the percentage of individuals in 20s and 30s categorized by the consumption of carbonated beverages. The percentage of individuals who consumed carbonated beverages was higher among those in 30s than among those in 20s. The percentage and standard error of participants in their 20s with no consumption, consumption once or less per month, once or less per week and twice or more per week were 2.9 ± 0.9%, 5.8 ± 1.4%, 6.5 ± 1.0%, and 7.1 ± 1.2%, respectively (*P* < 0.05). The percentage and standard error of participants in their 30s with no consumption, consumption once or less per month, consumption once or less per week and consumption twice or more per week were 14.7 ± 1.4%, 15.4 ± 1.5%, 17.5 ± 1.6%, and 20.0 ± 1.9%, respectively (*P* < 0.05).

Table 2 shows adjusted odds ratios and their 95% confidence intervals from multiple logistic regression analyses for the individuals with periodontal treatment needs. The odds ratios of the percentage of individuals with periodontal treatment needs tended to increase with the consumption of carbonated beverages. Adjusted odds ratios and their 95% confidence intervals for the individuals with consumption once or less per month, once or less per week and twice or more per week were 1.109(0.804,1.528), 1.404(1.035,1.906), and 1.466(1.059,2.029), respectively (*P* for trend < 0.05).

**Table 2:**
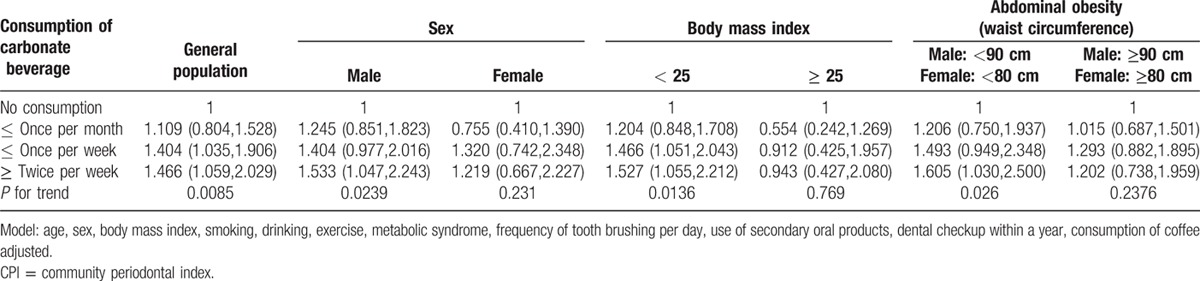
Adjusted odds ratio, 95% confidence interval, and *P* value of periodontal treatment needs (CPI ≥ 3) in the multivariate logistic regression model for consumption of carbonated beverages.

Adjusted odds ratios and their 95% confidence intervals of the male participants were 1.245(0.851, 1.823), 1.404(0.977,2.016), and 1.533(1.047,2.243) for once or less per month, once or less per week and twice or more per week, respectively (*P* for trend < 0.05). Adjusted odds ratios and their 95% confidence intervals regarding female individuals were 0.755(0.410,1.390), 1.320(0.742,2.348), and 1.219(0.667,2.227) for consumption once or less per month, once or less per week, and twice or more per week, respectively (*P* for trend > 0.05). In a subgroup analysis, adjusted odds ratios and their 95% confidence intervals of the individuals having body mass index < 25 were 1.204(0.848,1.708), 1.466(1.051,2.043), and 1.527(1.055,2.212), for consumption once or less per month, once or less per week and twice or more per week respectively (*P* for trend < 0.05). Adjusted odds ratios and their 95% confidence intervals of the individuals having body mass index ≥ 25 were 0.554(0.242,1.269), 0.912(0.425,1.957), and 0.943(0.427,2.080) for once or less per month, once or less per week, and twice or more per week, respectively (*P* for trend > 0.05). In another subgroup analysis regarding abdominal obesity, adjusted odds ratios and their 95% confidence intervals regarding individuals having waist circumference < 90 cm for males or < 80 cm for females were 1.206(0.750,1.937), 1.493(0.949,2.348), and 1.605(1.030,2.500) for consumption once or less per month, once or less per week, and twice or more per week, respectively (*P* for trend < 0.05). Adjusted odds ratios and their 95% confidence intervals regarding individuals having waist circumference ≥ 90 cm for males or ≥ 80 cm for females were 1.015(0.687,1.501), 1.293(0.882,1.895), and 1.202(0.738,1.959) for consumption once or less per month, once or less per week, and twice or more per week, respectively (*P* for trend > 0.05).

## 4 Discussion

This study demonstrated that the risk of periodontal disease was positively associated with consumption of carbonated beverages among Korean adults. This association between consumption of carbonated beverages and periodontal disease was independent of various potential confounding factors including systemic diseases and oral health behaviors. A subgroup analysis revealed that in individuals with body mass index < 25, or waist circumference < 90 cm for males or < 80 cm for females, the prevalence of periodontal disease increased with higher consumption of carbonated beverages.

Previous report showed that the consumption of carbonated beverages was associated with systemic diseases. High consumption of sugar-sweetened beverages was suggested to be associated with obesity and other adverse health issues.^[17,18]^ It was suggested that sugar-sweetened carbonated beverages may contribute to weight gain partly by incomplete compensation for energy at subsequent meals following consumption of liquid calories.^[19]^ Observational studies also indicated that consumption of sugar-sweetened soft drinks could promote obesity owing to low-satiating effects.^[20]^ The high consumption of carbonated beverages may be of great public health significance for girls and women because of their proneness to osteoporosis in later life.^[21,22]^ Children and adolescents with high intakes of carbonated beverages could be at higher risk of hypertension and metabolic syndrome.^[23]^ Soft drink consumption showed an association with nonalcoholic fatty liver disease independent of metabolic syndrome.^[24]^ Significant associations were noted between the consumption of carbonated beverages and poor self-reported academic grades, inadequate sleep, sedentary behaviors, and cigarette smoking.^[17]^

Carbonated beverages usually contains various compounds including caffeine, phosphate compounds, and potassium.^[3]^ Over 60% of soft drinks sold in the United States contain caffeine, a mildly addictive psychoactive chemical.^[25]^ It should be highlighted that high rates of consumption of caffeinated soft drinks more likely reflect the mood-altering and physical dependence-producing effects.^[25,26]^ Carbonated beverages often contain phosphate compounds, but the phosphate level is not usually provided in the ingredient list.^[27]^ Significantly higher urinary calcium (adjusted using urinary creatinine) excretion was found after consuming carbonated beverages compared to the fasting baseline level.^[27]^ The chronic consumption of large amounts of soft drinks may adversely affect potassium homeostasis and result in hypokalemia, leading to osmotic diarrhea, osmotic diuresis, and hyperinsulinemia.^[28,29]^

In the dental field, consumption of carbonated beverages often is associated with damage to the human dentition, the most common of which is dental erosion.^[30,31]^ Carbonated beverage of cola may stain the dental restoration material of glass-ionomer and composite resin.^[32,33]^ Carbonated beverage of cola may also cause increased release of metal ions (nickel) from orthodontic appliances.^[34]^ Positive correlations between occurrence of fungi and consumption of sweetened carbonated drinks were presented previously.^[35]^

The mechanisms for the association of the consumption of sugar-sweetened beverages with periodontitis may be explained partially by the following. Consumption of sugar-sweetened carbonated beverages was suggested to increase chronic inflammation.^[36]^ Sugar-sweetened carbonated beverages may contribute to a high dietary glycemic load, leading to inflammation, insulin resistance, and impaired beta-cell function.^[19]^ It was shown that daily intake of sugar-sweetened carbonated beverages for 6 months increased ectopic fat accumulation and lipids (liver fat, skeletal muscle fat, visceral fat, blood triglycerides, and total cholesterol).^[37]^ The fructose fraction of sugar-sweetened beverages may worsen accumulation of visceral fat, increased hepatic lipogenesis, and hypertension.^[19]^ Experimental evidence was obtained from animal models that sugar-sweetened soft drinks may play a role in the development of metabolic disorders in later life.^[38]^ These inflammatory changes due to consumption of carbonated beverages may have contributed to higher prevalence of periodontal disease.

The mechanisms for the association of the consumption of sugar-sweetened beverages with obesity are only partly understood.^[39]^ A previous report showed that a positive association between sugar-sweetened soft drink intake and type 2 diabetes risk was attenuated by adjustment for the body mass index.^[40]^ Consumption of sugar-sweetened beverages including carbonated beverages was positively related to insulin resistance and higher plasma leptin concentrations in nonoverweight women.^[39]^ A subgroup analysis regarding body mass index and waist circumference showed that those with body mass index < 25 and waist circumference < 90 cm for males or < 80 cm for females were more susceptible to periodontal disease. Thus, it can be suggested that individuals without obesity may benefit more from reduction of sugar-sweetened carbonated beverage consumption.

This phenomenon may partially be explained by the obesity paradox.^[41,42]^ Previous study showed that participants with moderate obesity was associated more strongly with a lower risk of mortality than with normal, underweight, and overweight groups in the general population of South Korea and this obesity paradox was prominent in not only the elderly but also individuals with chronic disease.^[43]^ It has also been suggested that obese patients tend to fare better after certain surgical procedures.^[42]^ Previous reports also demonstrated that the high body mass index may represent an optimal physical and nutritional state for protecting against catabolic diseases.^[43]^ Low muscle mass can be a poor prognostic indicator of mortality, and the body mass index was positively correlated with muscle mass or lean body mass, especially in the elderly.^[43,44]^ A true protective effect of obesity may be possible, mediated through differences in the immune response and more metabolic reserves.^[41]^ A longitudinal study or a clinical trial seems necessary to ascertain the associations between the consumption of carbonated beverages and periodontal disease.

This study showed the sex differences of odds ratios of periodontal treatment needs for consumption of carbonated beverages. Previous reports have also shown sex differences.^[39,45]^ Consumption of sugar-sweetened beverages including carbonated beverages was positively related to insulin resistance and higher plasma leptin concentrations in men.^[39]^ Conversely, it was shown that the increase in consumption of sugar-sweetened soft drinks from childhood to adulthood was directly associated with body mass index in adulthood in women but not in men.^[45]^ Further studies seems necessary to elucidate the mechanisms underlying the sex differences.

This study is based on a nationally representative sample of Koreans, and this may provide sufficient power for the investigation of the relationships between carbonated beverage consumption and periodontal disease and the potential influence of various relevant confounding factors.^[9,46]^ However, it also should be mentioned that limitations lie in the cross-sectional design of the present study and that the design cannot explain the causal relationship between the consumption of carbonated beverages (the exposure) and periodontal disease (the outcome).^[11,47]^ Second, the data regarding the history of consumption of carbonated beverages were collected by self-report, and the collection of these data was therefore subject to recall bias and may have been characterized by disparities between data details and the actual history.^[48,49]^ Third, this study used partial-mouth recording protocols of CPI because it was not feasible to conduct the traditional full-mouth examination due to limited resources including manpower, funds, and time.^[13,50]^ and due to the limitation of CPI, the prevalence of periodontal disease has been underestimated.^[50]^

In conclusion, consumption of carbonated beverages was positively associated with the risk of periodontal disease in Korean adults. In a subgroup analysis, the individuals consuming more carbonated beverages with body mass index < 25, or waist circumference < 90 cm for males or < 80 cm for females were more likely to have periodontal disease. Therefore, consumption of carbonated beverages may be considered to be an independent risk indicator of periodontal disease and we suggest that periodontal health of nonobese individuals may benefit from reduction of carbonated beverage consumption.

## Acknowledgments

The authors thank the Korea Centers for Disease Control and Prevention for providing the data.
